# Efficient Capture of Infected Neutrophils by Dendritic Cells in the Skin Inhibits the Early Anti-Leishmania Response

**DOI:** 10.1371/journal.ppat.1002536

**Published:** 2012-02-16

**Authors:** Flavia L. Ribeiro-Gomes, Nathan C. Peters, Alain Debrabant, David L. Sacks

**Affiliations:** 1 Laboratory of Parasitic Diseases, National Institute of Allergy and Infectious Diseases, National Institute of Health, Bethesda, Maryland, United States of America; 2 Division of Emerging and Transfusion Transmitted Diseases, OBRR, CBER, U.S. Food and Drug Administration, Bethesda, Maryland, United States of America; Imperial College London, United Kingdom

## Abstract

Neutrophils and dendritic cells (DCs) converge at localized sites of acute inflammation in the skin following pathogen deposition by the bites of arthropod vectors or by needle injection. Prior studies in mice have shown that neutrophils are the predominant recruited and infected cells during the earliest stage of *Leishmania major* infection in the skin, and that neutrophil depletion promotes host resistance to sand fly transmitted infection. How the massive influx of neutrophils aimed at wound repair and sterilization might modulate the function of DCs in the skin has not been previously addressed. The infected neutrophils recovered from the skin expressed elevated apoptotic markers compared to uninfected neutrophils, and were preferentially captured by dermal DCs when injected back into the mouse ear dermis. Following challenge with *L. major* directly, the majority of the infected DCs recovered from the skin at 24 hr stained positive for neutrophil markers, indicating that they acquired their parasites via uptake of infected neutrophils. When infected, dermal DCs were recovered from neutrophil depleted mice, their expression of activation markers was markedly enhanced, as was their capacity to present Leishmania antigens *ex vivo*. Neutrophil depletion also enhanced the priming of *L. major* specific CD4^+^ T cells *in vivo*. The findings suggest that following their rapid uptake by neutrophils in the skin, *L. major* exploits the immunosuppressive effects associated with the apoptotic cell clearance function of DCs to inhibit the development of acquired resistance until the acute neutrophilic response is resolved.

## Author Summary

Prior studies in mice have shown that the inoculation of *Leishmania major* into the skin by sand fly bite or by needle provokes a massive recruitment of neutrophils that take up the parasite, and that this response somehow suppresses immunity since neutrophil depletion results in better control of the infection. We investigated how neutrophils recruited to the injection site might interact with and suppress the function of dendritic cells (DCs) in the skin. Infected neutrophils recovered from the skin expressed increased levels of apoptotic markers compared to uninfected neutrophils, and were efficiently taken up by dermal DCs when injected back into the skin. When dermal DCs were permitted to take up parasites in the absence of neutrophils, their expression of activation markers and their ability to present Leishmania antigens were enhanced. Neutrophil depletion also enhanced the activation of Leishmania specific CD4^+^ T cells *in vivo.* The results suggest that for insect borne pathogens like Leishmania that provoke a strong inflammatory response at the site of infection, the immunosuppressive effects associated with the apoptotic cell clearance function of DCs will inhibit the early development of immunity.

## Introduction

Leishmaniasis is a vector-borne disease initiated by the bite of an infected sand fly. Based on exhaustive findings in the murine model of cutaneous leishmaniasis due to *Leishmania major*, the clinical course of disease is thought to depend on the balance of activating cytokines, produced largely by Th1 cells, and deactivating cytokines, produced largely by Th2 cells and subsets of regulatory T cells [1]. Even in genetically resistant C57BL/6 mice, however, that develop self-limiting lesions due to a strongly polarized Th1 response, the early growth of the parasite is unrestrained, suggesting that innate killing mechanisms and the development of acquired resistance are avoided or delayed [2]. There is evidence that the acute neutrophilic response is itself critical to the early establishment of infection in the skin [3], [4]. Inoculation of *L. major* by the bite of a sand fly, or by needle injection, induces an intense infiltration of neutrophils that phagocytose the majority of parasites but fails to kill them, and neutrophil depletion prior to sand fly challenge leads to more rapid parasite clearance [5]. The manner in which the acute neutrophilic response inhibits the development of immunity to *L. major* infection is not understood.

Neutrophils and DCs are normally located in distinct anatomical compartments, but converge at sites of inflammation in response to infection or tissue injury. The essential function of neutrophils in phagocytosis and killing of bacteria and in tissue repair is well described [6], [7]. Their additional role in modulating the adaptive response is suggested by their ability to release chemokines, cytokines, and anti-microbial peptides, [8], [9], and by more recent findings suggesting that activated neutrophils can deliver both activation signals and microbial antigens to DCs [10], [11]. By contrast, engulfment of apoptotic cells, including neutrophils, by DCs under steady state conditions has been shown to suppress DC maturation and is thought critical to the maintenance of peripheral tolerance [12]–[14]. Thus, the immunologic outcome of neutrophil - DC interactions may vary depending on the activation state of the neutrophils, their type of cell death, and the presence or absence of additional danger signals in the microenvironment in which these encounters occur.

Importantly, the cross-talk between neutrophils and DCs has not been investigated in the context of any vector borne pathogen for which the co-localization of these cells at the site of transmission by bite or injection by needle in the skin is apt to be especially pronounced. In the present studies, we have monitored the sequence of inflammatory events following infection with *L. major* in the mouse ear dermis. We provide clear evidence that dermal DCs are preferentially infected via their capture of parasitized neutrophils in the skin, and that the Leishmania specific CD4^+^ T cell response is compromised until the acute neutrophilic response is resolved.

## Results

### Changes in dermal myeloid cell populations following *L. major* infection

**Figure 1:**
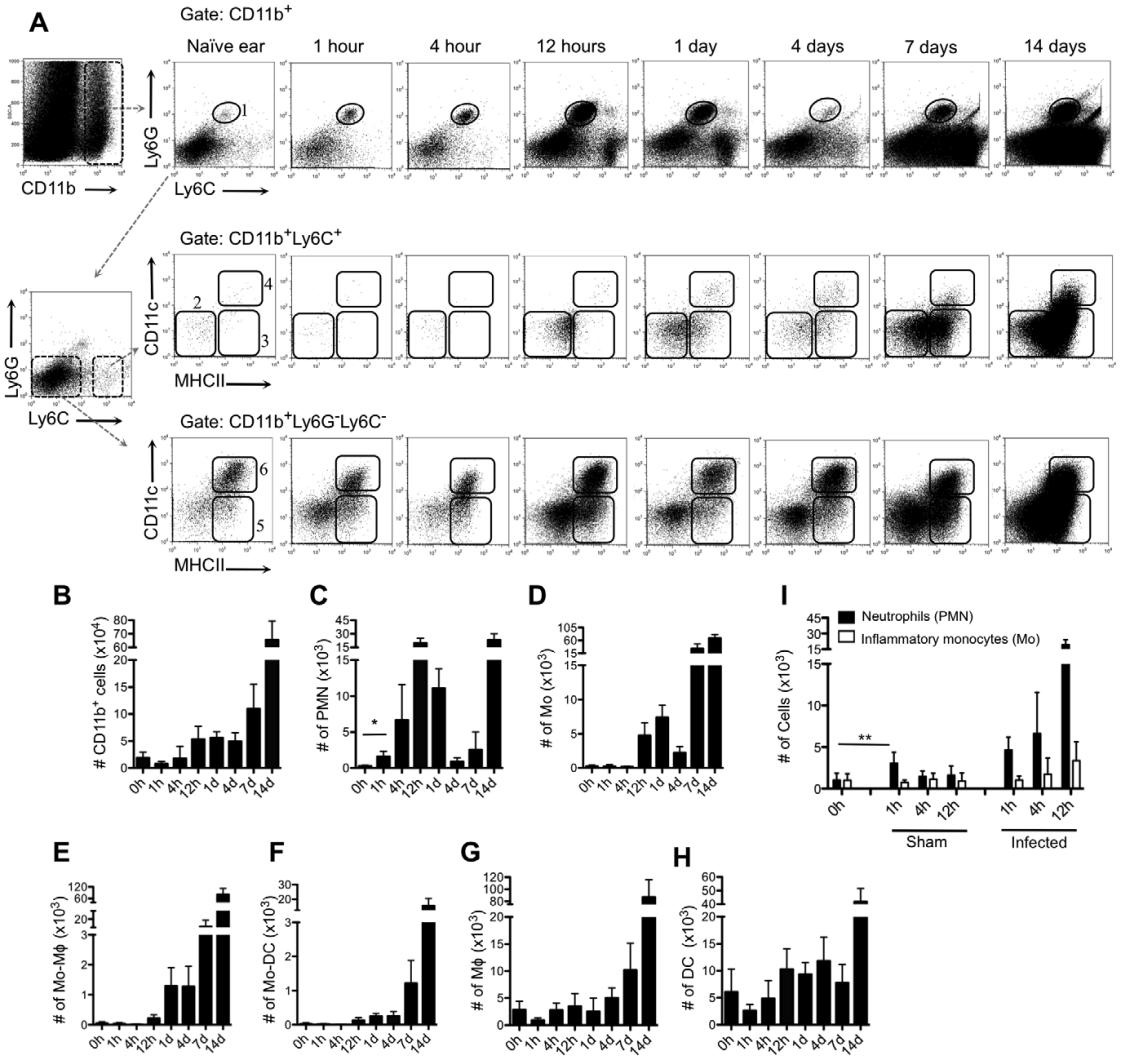
Kinetics of myeloid cell recruitment following i.d. inoculation of *L. major*. (A) Representative dot plots of ear-derived dermal cells recovered at different times after i.d. infection with 2×10^5^ *Lm*-RFP. Subpopulations of CD11b**^+^** myeloid cells are defined by the following markers: Ly6C^int^Ly6G^+^ neutrophils (PMN; region 1); Ly6C^hi^Ly6G^−^ inflammatory monocytes (Mo; region 2) Ly6C^hi^Ly6G^−^CD11c^−^MHCII^+^ monocytes/macrophages (Mo-Mφ; region 3); Ly6C^hi^Ly6G^−^CD11c^+^MHCII^+^ monocyte-derived dendritic cells (Mo-DC; region 4); Ly6C^−^Ly6G^−^CD11c^−^MHCII^+^ macrophages (Mφ; region 5); Ly6C^−^Ly6G^−^CD11c**^+^**MHCII**^+^** dendritic cells (DC; region 6). (B–H) Changes in the total number of CD11b**^+^** cells, PMN, Mo, Mo-Mφ, Mo-DC, Mφ, and DC per ear. Values shown are the mean numbers of cells per ear +/− 1 s.d., 6–8 ears at each time point, pooled data from two independent experiments. * *p* = 0.0006. (I) Total number of ear-derived neutrophils and inflammatory monocytes in sham and *Lm*-RFP injected mice. Values shown are the mean numbers of cells per ear +/− 1 s.d., 6–8 ears at each time point, pooled data from two independent experiments. ** *p* = 0.0174.

We investigated the sequence of local inflammatory responses and identified the cells harboring *L. major* following injection of *Lm*-RFP metacyclic promastigotes (2×10^5^) in the ear dermis of C57BL/6 mice. Myeloid populations were identified as CD11b^+^ cells, and further classified based on their expression of additional markers (Figure 1A) as follows: neutrophils (Ly6C^int^Ly6G^+^, region 1); inflammatory monocytes (Ly6C^hi^Ly6G^−^CD11c^−^MHCII^−^, region 2); monocytes/macrophages (Ly6C^hi^Ly6G^−^CD11c^−^MHCII^+^, region 3); monocyte-derived DCs (Ly6C^hi^Ly6G**^−^**CD11c**^+^**MHCII**^+^**, region 4); dermal DCs (Ly6C**^−^**Ly6G**^−^**CD11c**^+^**MHCII**^+^**, region 6); and dermal macrophages (Ly6C**^−^**Ly6G**^−^**CD11c**^−^**MHCII**^+^**, region 5). The cells in region 5 were uniformly F4/80**^+^** (data not shown). The CD11b**^+^** cells recovered from naïve ears included few neutrophils and inflammatory monocytes, and relatively greater numbers of dermal DCs and macrophages. The total number of CD11b**^+^** cells recovered from the infected ears increased slowly over the first week, and expanded dramatically over the second week (Figure 1B). A prominent and transient neutrophil infiltrate accounted for the earliest increase in myeloid cells in the site, beginning at 1 hr, peaking at 12 hr, and dropping markedly between 1- 4 days (Figure 1C). Interestingly, neutrophils were found infiltrating the site again at day 7, and by day 14 their numbers exceeded the peak numbers observed during the first wave of the neutrophilic response. Comparison of *L. major* infected and sham injected mice demonstrated that at 1 hr the initial neutrophil infiltrate was induced, at least in part, by the tissue injury associated with the needle injection. At subsequent time points, however, the recruitment was dependent on the infectious status of the inoculum (Figure 1I).

The increase in the number of inflammatory monocytes (Figure 1D) lagged slightly behind the neutrophil response, beginning at 12 hr and peaking at 24 hr. Similarly to the neutrophils, their numbers dropped markedly by 4 days but began to increase again by day 7. Very few of the CD11b^+^Ly6C^hi^Ly6G^−^ cells recovered from the site during the first week of infection were MHCII^+^ or CD11c^+^ (Figure 1F), of note because of recent findings implicating monocyte-derived DCs formed at the infection site as crucial to the induction of protective immunity during the active stage of disease [15]. The number of macrophages and DCs remained relatively unchanged from steady state conditions until 7 days post-infection, marking the onset of their massive accumulation in the site (Figure 1G and 1H).

### Analysis of *L. major* infected dermal cell subpopulations

**Figure 2:**
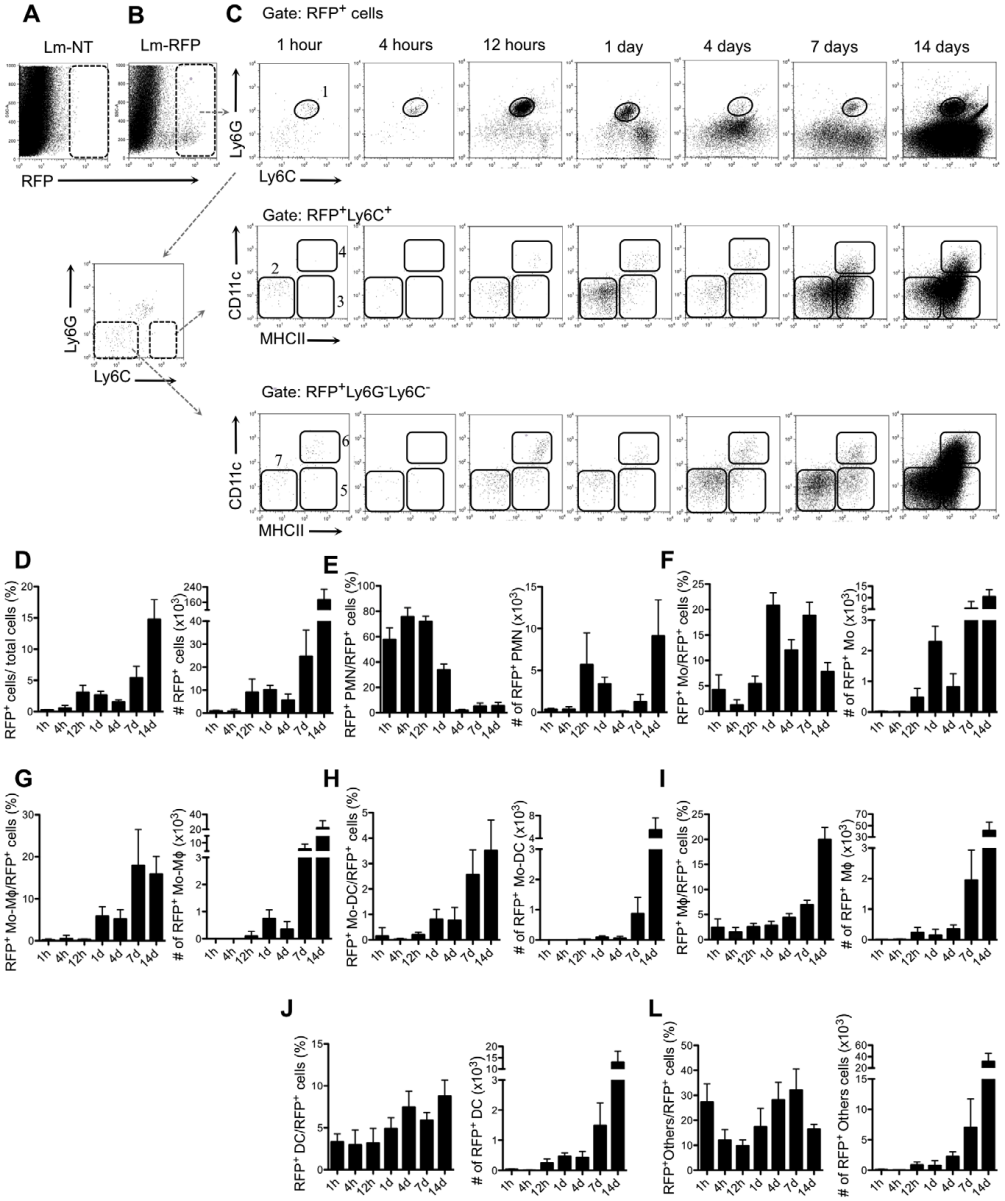
Kinetics of inflammatory cell subsets infected by *L. major*. (A–B) SSC/RFP dot plots of ear-derived cells 1 hr post-infection with 2×10^5^ *L. major-* vector control (*Lm*-NT) (A) or *L. major*-RFP (*Lm*-RFP) (B). (C) Representative dot plots of RFP^+^ ear-derived cell subsets at different time points after i.d. infection with *Lm*-RFP. Regions 1–6 define the same subsets of CD11b**^+^** myeloid cells as defined in the corresponding regions in Figure 1A. Region 7 (others) delineates a poorly defined Ly6G**^−^**Ly6C**^−^**CD11c**^−^**MHCII**^−^** population that includes CD11b**^+^** and CD11b**^−^** cells. (D) Changes in the RFP**^+^** cells expressed as a percentage of total ear dermal cells, and their absolute numbers recovered at each time point. (E–L) Changes in the subsets of RFP**^+^** cells expressed as a percentage of the total RFP**^+^** cells, and their absolute numbers recovered at each time point. Values shown are the mean numbers of cells per ear +/− 1 s.d., 6–8 ears at each time point, pooled data from two independent experiments.

By analyzing the total population of RFP^+^ gated cells, we could follow the subsets of infected cells in the injection site over time (Figure 2A–C). Regions 1–6 define to the same subsets of myeloid cells as the corresponding regions in figure 1A, and in each case their CD11b expression was confirmed (data not shown). By contrast, many of the infected cells in region 7 were CD11b^−^, and their identity was not established using additional markers. Considering the total population of RFP**^+^** cells (Figure 2D), low numbers were recovered at 1 and 4 hr which significantly increased between 4–12 hr and dramatically increased between 7–14 days (Figure 2D). In Figures 2E–L, the infected subsets are expressed both as a percentage of the total infected cells and their absolute numbers recovered from the ear dermis at each time point. Neutrophils were the predominant infected cells during the first 1–12 hrs (Figure 2E). At 12 hrs, 72% of the infected cells were neutrophils, with the remainder inflammatory monocytes, macrophages, DCs and other populations of CD11b^+^ and CD11b^−^ cells. At 24 hr, neutrophils still represented approximately 32% of the total RFP^+^ cells. By day 4, the percentage of neutrophils in the RFP^+^ gate had dropped to fewer than 1%. Interestingly, their numbers began to increase again by day 7, and by day 14, the absolute number of infected neutrophils in the site exceeded the peak numbers observed during the first wave, although they remained <5% of the total population of infected cells. The inflammatory monocytes in the RFP^+^ gate also demonstrated two phases of recruitment, the first peaking at day 1 when they represented 22% of the total RFP^+^ cells, and the second at day 7 (Figure 2F). Their absolute numbers were greatest at day 14, again reflecting the massive expansion in the total number of infected cells at this time point. Very few of the RFP^+^ inflammatory monocytes recovered during the first 4 days were MHCII^+^ or CDllc^+^, while at 7 and 14 days, the majority of the RFP^+^ Ly6C^hi^Ly6G^−^ cells were MHCII^+^ and CD11c^−^ (Figure 2G), reflecting the early stage of their differentiation to macrophages in the site. By day 14, appreciable numbers of infected monocyte-derived DCs (Figure 2H) were recovered, though they still represented only around 4% of the total RFP^+^ cells. By contrast, the infected macrophages (Figure 2I), expressed both as a percentage and absolute number of infected cells, started to increase at 4 days, and accounted for up to 20% of the total RFP^+^ cells at 14 days. The RFP^+^ dermal DCs remained few in number and <5% of the RFP^+^ cells over the first 24 hr, and while they remained a low percentage of the RFP^+^ cells at later time points, their absolute numbers increased markedly at 7 days and especially at 14 days post-infection (Figure 2J).

In summary, our detailed analysis of infected cells in the *L. major* loaded dermis confirmed that neutrophils rapidly infiltrating the site represent the vast majority of infected cells over the first 12 hr, with the infections transitioning to inflammatory monocytes, and finally to monocyte derived macrophages and DCs during the active stage of disease.

### Uptake of parasitized neutrophils by DCs in the skin

**Figure 3:**
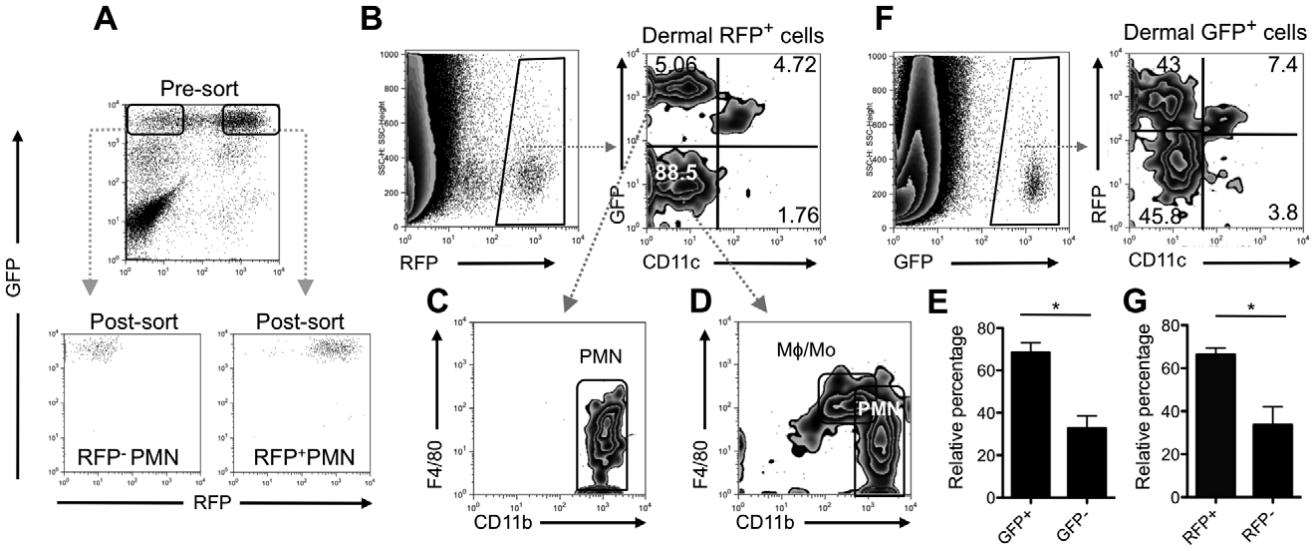
Uptake of infected neutrophils by dermal DCs. (A) LYS-eGFP^hi^ neutrophils recovered from the ear dermis 12 hrs after infection with 2×10^6^ *Lm*-RFP metacyclic promastigtoes were sorted to obtain uninfected RFP**^−^** and infected RFP**^+^** neutrophils. (B–D) Representative dot plots of gated RFP**^+^** dermal cells recovered from a single ear of a C57BL/6 mouse 4 hr after i.d. injection of 2.5×10^4^ RFP**^+^** neutrophils, and analyzed for their expression of eGFP, CD11c, F4/80 and CD11b. Quadrant values are the percentage of total RFP**^+^** cells. (E) RFP**^+^** DCs that are eGFP**^+^** or eGFP**^−^** (mean percentage +/− 1 s.d.), calculated from the analysis shown in (B) involving 4 independent experiments, 1–2 ears per experiment; * *P*<0.0001. (F) Representative dot plots of gated eGFP**^+^** dermal cells recovered from a single ear of a C57BL/6 mouse 4 hr after i.d. injection of 2.5×10^4^ of uninfected RFP**^−^** and 2.5×10^4^ infected RFP**^+^** neutrophils and analyzed for their expression of RFP and CD11c. Quadrant values are the percentage of total eGFP**^+^** cells. (G) Total eGFP**^+^** DC that are RFP**^+^** or RFP**^−^** (mean percentage +/− 1 s.d.), calculated from the analysis shown in (F) involving 4 independent experiments, 1 ear per experiment; * *P*<0.0001.

Given their predominance both as the earliest infiltrating and parasitized cells in the injection site, we investigated the influence of neutrophils on the subsequent program of infection and immune response. *In vitro* studies have suggested that macrophages can acquire *L. major* by phagocytosing infected, apoptotic neutrophils [16], [17]. To investigate the fate of infected neutrophils and their internalized parasites, *Lm*-RFP metacyclic promastigotes were injected into the ears of LYS-eGFP mice [18], in which neutrophils (CD11b^hi^Gr-1^hi^F4/80^−^MHCII^−^), including those recovered from the skin, are eGFP^hi^ [5]. eGFP^hi^RFP^+^ infected neutrophils were purified by cell sorting (Figure 3A) and injected into the ears of C57BL/6 mice. Analysis of a stained, cytospin preparation of the sorted cells just prior to injection indicated that approximately 30% of the parasites had already been released from the neutrophils during the 4–5 hr collection. Four hours after injection, the vast majority of the RFP^+^ cells recovered from the ear (90%) were found in an eGFP^−^ population (Figure 3B), suggesting that in addition to the free parasites present in the inoculum, most of the remaining parasites were released from the infected neutrophils and available to be taken up by host cells in the skin. These cells were CD11c^lo^, and their F4/80 and CD11b expression indicated that they were endogenous macrophages/monocytes or neutrophils (Figure 3D). Of the RFP^+^ cells that retained their eGFP fluorescence, approximately half appeared to be the injected population of intact, infected neutrophils (Figure 3C). The remaining RFP^+^eGFP^+^ cells were CD11c^+^, suggesting that the capture of infected neutrophils in the skin was largely accomplished by DCs. Of the total number of CD11c^+^RFP^+^ cells, 68% were eGFP^+^ (Figure 3E), suggesting that most of the DCs acquired their parasites *via* uptake of infected neutrophils. Of note, the eGFP fluorescence in these cells was reduced relative to the starting population of infected neutrophils. To rule out the possibility that the eGFP^hi^RFP^+^ infected neutrophils could have differentiated into CD11c^+^ cells, or that a small contaminating population of CD11c^+^ cells in the purified eGFP^hi^RFP^+^ infected neutrophils was responsible for the RFP^+^eGFP^+^CD11c^+^ cells observed in figure 3B, we sorted eGFP^hi^RFP^+^ infected neutrophils (donor, CD45.2), and injected them into the ears of B6SJL mice (host, CD45.1). Analysis of CD45.1 expression on the subpopulations of RFP^+^ cells indicated that virtually all of the RFP^+^eGFP^+^CD11c^+^ cells were CD45.1^+^, ruling out their donor origin (Figure S1).

To investigate whether DCs might favor engulfment of infected neutrophils over uninfected neutrophils in the skin, equal numbers of eGFP^hi^RFP^−^ uninfected and eGFP^hi^RFP^+^ infected neutrophils (Figure 3A) were co-injected into the ears of C57BL/6 mice. The analysis of eGFP^+^ gated cells recovered four hours later confirmed that DCs are able to take up neutrophils *in vivo*, representing approximately 11% of the eGFP^+^ cells (Figure 3F). Importantly, and despite their exposure to equivalent numbers of infected and uninfected neutrophils, an average of 66% of the CD11c^+^eGFP^+^ cells were RFP^+^ (Figure 3F and G), indicating that the dermal DCs favored the uptake of the infected neutrophils.

**Figure 4:**
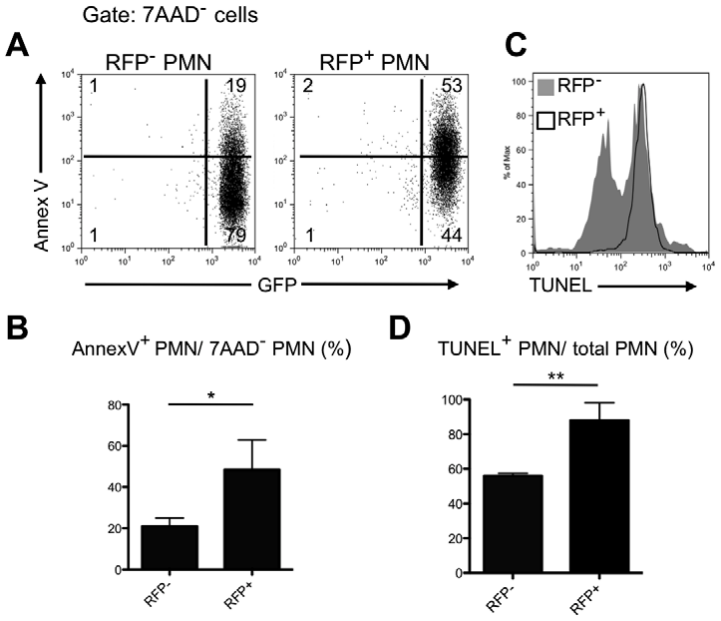
Uptake of *L. major* leads to accelerated apoptosis of neutrophils in the skin. (A) Representative dot plots of sorted RFP**^−^** or RFP**^+^** LYS-eGFP^hi^ neutrophils recovered from the ear dermis 12 hrs after infection with 2×10^6^ *Lm*-RFP metacyclic promastigotes and stained with annexin V-APC after gating on 7-AAD**^−^** cells. Quadrant values show the percentage of total gated cells. (B) Annexin-V**^+^** 7-AAD**^−^** cells (mean percentage +/− 1 s.d.) calculated from 3 independent experiments; * *p* = 0.034. (C) Representative histogram plot of RFP**^−^** (gray filled) and RFP**^+^** (black line) neutrophils subjected to TUNEL staining. (D) TUNEL**^+^** cells (mean percentage +/− 1 s.d.) calculated from 2 independent experiments; ** *p* = 0.047.

The capture of mammalian cells by DCs and others phagocytes is a common event during tissue remodeling and at infection sites, where cells dying by apoptosis expose signals that are recognized for engulfment. The best studied ‘eat me’ signal on apoptotic cells is phosphatidylserine (PtdSer) whose outer membrane exposure can be quantified by staining with Annexin V. When neutrophils were recovered from the ear dermis of LYS-eGFP mice 12 hr after infection, 53% of the infected neutrophils compared to 19% of the uninfected neutrophils were Annexin V**^+^** (Figure 4A and B). TUNEL staining confirmed a higher degree of apoptosis in the infected population of neutrophils recovered from the injection site (Figure 4C and D). These findings suggest that the uptake of *L. major* leads to accelerated apoptosis and earlier exposure of PtdSer on neutrophils infiltrating the injection site, which may favor their recognition and capture by DCs in the skin.

### Infected DCs recovered from the skin express neutrophil markers

**Figure 5:**
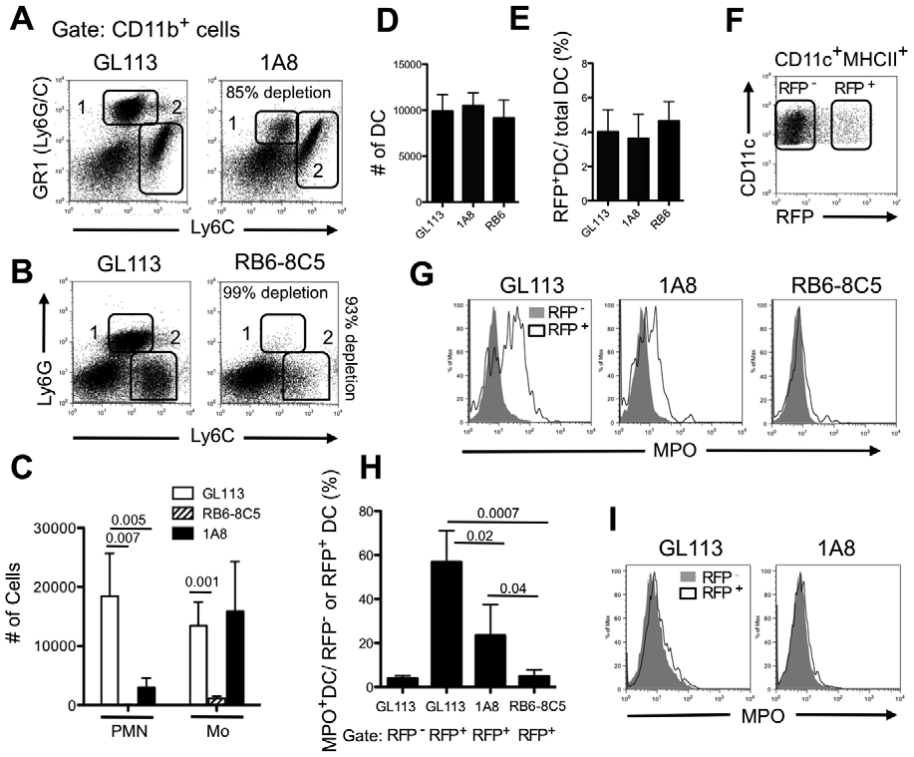
Dermal DCs infected with *L. major* display neutrophil markers. Mice were treated with control (GL113) or neutrophil-depleting (RB6-8C5 or 1A8) monoclonal antibodies 1 d before challenge in the ear dermis with 2×10^5^ *Lm*-RFP metacyclic promastigotes. (A) Representative dot plots of CD11b^+^GR-1^hi^Ly6C^int^ neutrophils (region 1) and CD11b^+^GR1^int^Ly6C^hi^ inflammatory monocytes (region 2) in the GL113 or 1A8 treated mice. (B) Representative dot plots of CD11b^+^Ly6G**^+^**Ly6C^int^ neutrophils (region 1) and CD11b^+^Ly6G**^−^**Ly6C^hi^ inflammatory monocytes in the GL113 or RB6-8C5 depleted mice (region 2). (C) Mean total number per ear of neutrophils (PMN) or inflammatory monocytes (Mo) in neutrophil depleted or control treated, infected mice, +/− 1 s.d., 4–6 ears per group pooled from 2 independent experiments. (D–E) Mean total number of DCs (CD11c^+^MHCII^+^) per ear (D) and the mean percentage of RFP**^+^** DCs per ear (E) 1 d after infection, +/− 1 s.d., 8–10 ears per group, pooled from 3 independent experiments. (F) Representative dot plot of CD11c**^+^**MHCII**^+^**-gated RFP**^−^** uninfected and RFP**^+^** infected dermal DCs, 24 hr after infection. (G) Representative histogram plots of MPO stained, RFP**^−^** (gray filled) and RFP**^+^** (black line) DCs 24 hr after infection. (H) Mean percentage of RFP**^−^** or RFP**^+^** DCs staining positive for MPO, +/− 1 s.d., 4 ears per group, pooled from two independent experiments. (I) Representative histogram plots of MPO stained, RFP**^−^** (gray filled) and RFP**^+^** (black line) DCs. Mice were treated with control or neutrophil-depleting antibodies 1 d before i.d. challenge and the dermal cells were analyzed 14 days after infection.

To investigate neutrophil - DCs interactions following injection of the parasite directly, we evaluated dermal DCs recovered from C57BL/6 mice 24 hr after infection with *Lm*-RFP parasites, and stained for neutrophil derived-myeloperoxidase (MPO) and elastase (NE). In addition, mice were treated with two neutrophil-depleting antibodies: the GR-1 specific antibody RB6-8C5, which recognizes an epitope shared by Ly6G and Ly6C, and the Ly6G specific antibody, 1A8. Administration of 1A8 one day before infection depleted 85% of the CD11b^+^GR1^hi^Ly6C^int^ neutrophils present in the ear dermis 24 hr after infection (Figure 5A and C). The remaining neutrophils showed lower GR1 staining, likely due to competition with the surface bound 1A8 antibody. The CD11b^+^GR1^int^Ly6C^hi^ population was unaffected. By contrast, and consistent with the prior reports [19], [20], the RB6-8C5 antibody depleted both neutrophils and a population of inflammatory monocytes (Figure 5B and C). Furthermore, the neutrophil depletion achieved using RB6-8C5 was virtually complete (99%). Neither reagent affected the total number of DCs recovered from the ear at 24 hr, or the number RFP^+^ DCs as a percentage of the total population of RFP^+^ cells (Figure 5D and E). Gating on RFP^+^ or RFP^−^ dermal DCs (Figure 5F), MPO staining on cells recovered from the control treated mice was observed in an average of the 58% of the RFP^+^ DCs, suggesting that the majority of the infected DCs acquired their parasites via uptake of infected neutrophils (Figure 5G and H). By contrast, only a low proportion (<5%) of the RFP^−^DCs were MPO^+^, although because far more RFP^−^DCs were recovered from the site compared to RFP^+^DCs (Figure 5F), the percentage of RFP^−^DCs staining for MPO was on average 60% of the total population of MPO^+^ DCs (data not shown). The intracellular MPO staining in the majority of the infected DCs was comparable to the MPO staining observed in the neutrophils themselves, and greater than the MPO staining observed in the inflammatory monocytes recovered from the site (Figure S2), reinforcing the conclusion that the acquisition of the MPO marker by infected DCs was due to their uptake of infected neutrophils. Importantly, the RFP**^+^** DCs recovered from the RB6-8C5 treated mice were virtually all MPO**^−^**, confirming that the uptake of parasites in the absence of neutrophils or inflammatory monocytes does not upregulate the expression of MPO in the DCs. The number of RFP^+^ DCs recovered from the 1A8 treated mice that stained for MPO was also significantly reduced, though an average of 24% of the cells were still MPO**^+^** cells, consistent with the incomplete neutrophil depletion using this antibody (Figure 5G and H). Staining for NE, while relatively weak compared with MPO, reinforced the MPO result in that the majority of the RFP^+^ DCs recovered from the non-depleted mice stained positive for NE (Figure S3). Finally, RFP^+^ DCs recovered from control treated mice 14 days after infection were mainly MPO**^−^** (Figure 5I), suggesting that following the resolution of the acute neutrophilic response, infected neutrophils were no longer the main source of parasite delivery for DCs in the skin. We further characterized the possible subsets of the RFP^+^ DCs recovered from the site based on their expression of Langerin and CD103. As reviewed [21], and confirmed in our analysis of the DCs recovered from the ear dermis 24 hr after infection, the DC subsets include Langerhans cells (LC) and migratory LC (CD11c^+^MHCII^+^Lang^+^CD103^−^), Langerin^+^ DC (CD11c^+^MHCII^+^Lang^+^CD103^+^), and Langerin^−^ DC (CD11c^+^MHCII^+^Lang^−^CD103^−^) (Figure S4). The RFP signal was associated exclusively with the Langerin**^−^** DCs.

### Enhanced activation and function of infected DCs recovered from neutrophil depleted mice

**Figure 6:**
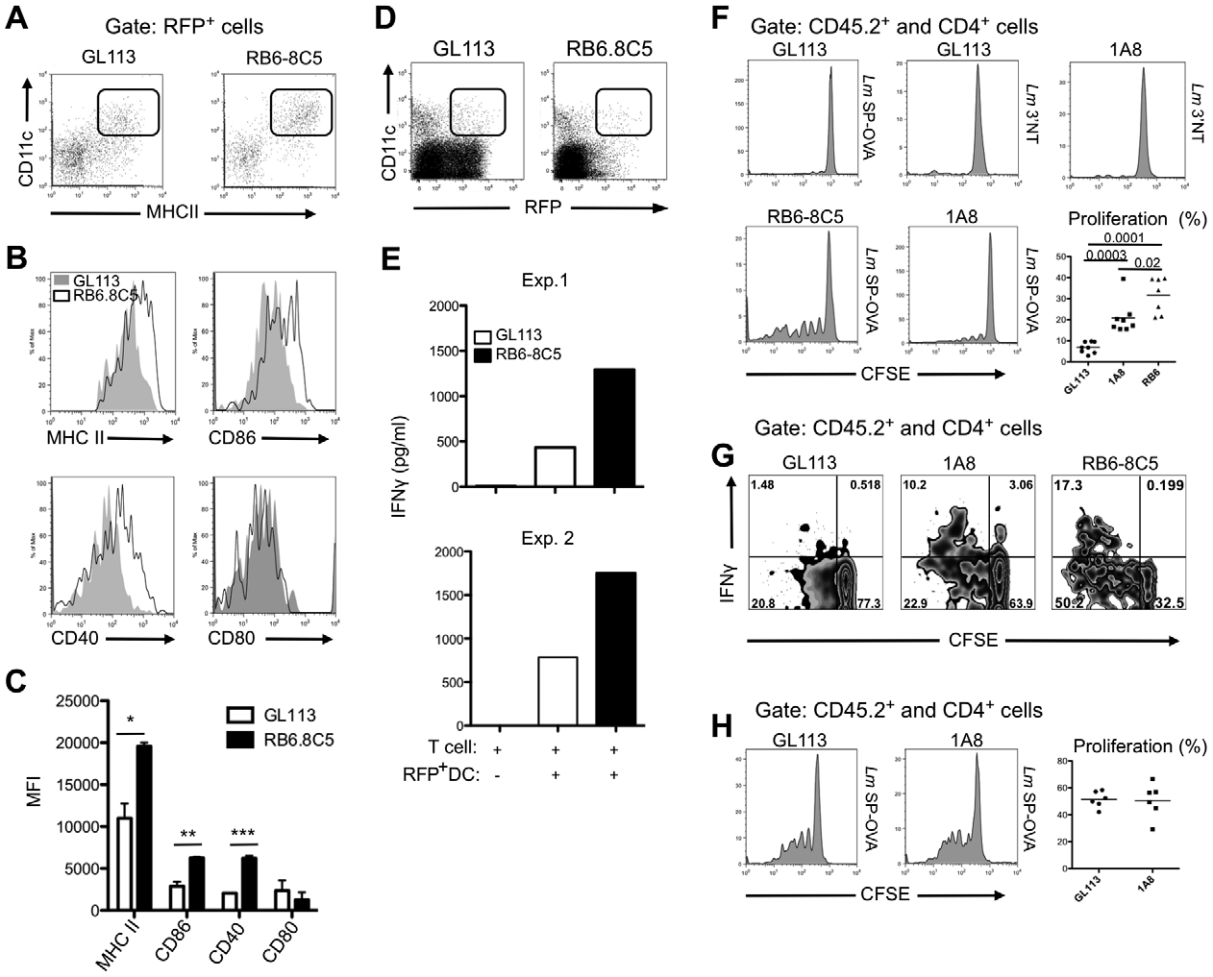
Effects of neutrophil depletion on the activation and function of dermal DCs *ex vivo*, and on CD4^+^ T cell priming *in vivo*. (A) Representative dot plots of RFP**^+^** DCs recovered 3 d after i.d. infection with 2×10^6^ *Lm*-RFP metacyclic promastigotes in mice treated with GL113 or RB6-8C5 1 d before challenge. (B) Representative histogram plots of RFP^+^CD11c^+^MHCII^+^ gated cells from control treated (gray filled) and RB6-8C5 treated (black line) infected mice stained for MHCII, CD86, CD40 and CD80. (C) Mean fluorescence intensity (MFI) of MHCII, CD86, CD40 and CD80 expression on RFP^+^CD11c^+^MHCII^+^ gated cells from control treated (white bars) and RB6-8C5 treated (black bars) infected mice; mean percentage +/− 1 s.d. calculated from 2 independent experiments; * *p* = 0.0214, ** *p* = 0.0134, *** *p* = 0.0025. (D) Representative dot plots showing the gates used for sorting of the RFP**^+^**CD11c^+^ dermal cells recovered from control and RB6-8C5 treated, infected mice. (E) IFN-γ levels in supernatants of T cells from healed, *L. major*-infected mice co-cultured for 3 d with sorted RFP^+^CD11c^+^ cells recovered from the ears of control treated or RB6-8C5 treated mice 3 days after i.d. infection with 2×10^6^ *Lm*-RFP metacyclic promastigotes. Two independent experiments are shown. (F–H) B6.SJL mice were treated with GL113, RB6-8C5 or 1A8 mAb followed 1 d later by i.d. inoculation with 10^5^ *Lm* SP-OVA or *Lm* 3′NT metacyclic promastigotes. CFSE-labeled CD4^+^ OT-II cells were adoptively transferred at the time of (F–G) or 2 weeks after infection (F). Shown are representative dot and histogram plots of IFN-γ expression and/or CFSE fluorescence of CD45.2**^+^**CD4**^+^** gated cells from the draining lymph nodes 6 days after transfer, and the proliferative response with means of CD4^+^ OT-II cells from individual mice, as defined by the percent of cells with reduced CFSE content. The data are pooled from two independent experiments.

To address whether neutrophils might modulate the antigen presentation functions of DCs during the early stages of infection, the expression of activation markers on infected DCs recovered from the ear dermis 3 days after infection in neutrophil-depleted (RB6-8C5) or control treated C57BL/6 mice was compared (Figure 6A–C). Expression of MHC class II, CD86 and CD40, but not CD80, was increased on RFP^+^ DCs recovered from the neutrophil depleted mice (Figure 6B and C). Functional studies involving these infected DCs required pooling of dermal cells from 10 mice (20 ears) for each treatment group in order to obtain a sufficient source of antigen and antigen presenting cells for the co-culture assays. Using CD11c^+^ RFP^+^ cells that were normalized for their RFP signals by cell sorting (Figure 6D), the infected DCs from neutrophil depleted mice were more efficient than the infected DCs from the control treated mice in activating Leishmania-primed T cells from healed mice to secrete IFN-γ, observed in two independent experiments (Figure 6E).

### Neutrophil depletion augments presentation of parasite-derived antigen *in vivo*

To evaluate the influence of neutrophils on CD4^+^ T cell priming to *L. major*- derived antigen *in vivo*, B6.SJL congenic mice were depleted of neutrophils 24 hr prior to infection with *L. major* SP-OVA or control 3′NT transgenic parasites in the ear. CFSE-labeled, naïve OT-II CD4^+^ T cells specific for OVA were adoptively transferred into the same recipients. Draining lymph nodes were harvested on day 6 and dilution of CFSE fluorescence was determined on CD45.2^+^ and CD4^+^ gated cells. Infection of control treated mice with *Lm* SP-OVA failed to induce OT-II proliferation above the background levels (6–7%) observed in control treated or neutrophil depleted mice infected with *Lm* 3′NT (Figure 6E). By contrast, mice treated with 1A8 and RB6-8C5 had an average of 20% and 34% of the gated cells in division, respectively (Figure 6F). We also assessed the ability of CD45.2^+^ OT-II CD4^+^ cells to produce IFNγ, IL-10 and IL-17, following ex-vivo restimulation with PMA/ionomycin for 4 hours in the presence of brefeldin-A. A percentage of proliferating CD45.2^+^ OT-II CD4^+^ cells from 1A8 and RB6-8C5 treated mice (29% and 25%, respectively) produced IFNγ (Figure 6G). Neither IL-10- nor IL-17A-producing T cells were detected (data not shown). The influence of early neutrophil depletion on CD4 priming was no longer apparent when OT-II cells were transferred 14 days post-infection with *Lm* SP-OVA (Figure 6H), at a time when infected DCs no longer harbored neutrophil markers (Figure 5I). Taken together, these findings suggest that the favored uptake of infected neutrophils by dermal DCs effectively prevents the activation of Leishmania-specific CD4^+^ T cells until the acute neutrophilic response is resolved.

## Discussion

We have recently described the efficient capture of *L. major* metacyclic promastigotes by neutrophils at the site of needle inoculation or infected sand fly bite, and the powerful effects of early neutrophil depletion in promoting rather than compromising host resistance to sand fly transmitted infection [5], [22]. The current studies provide an underlying mechanism to explain the immunomodulatory role of neutrophils in the *L. major* loaded dermis. Under steady state conditions, DCs are strategically positioned in peripheral and lymphoid tissues to sense microorganisms and endogenous stress signals, including apoptotic cells. Neutrophils, by contrast, are present mainly within the blood, and circulate in a non-activated state with a half-life of 6–7 hrs. Following inoculation of *L. major* into the skin by needle or by the bite of an infected sand fly, the parasites are taken up by neutrophils that are rapidly recruited to and accumulate with DCs at the injured site. We observed that phagocytosis of *L. major* significantly accelerated the rate of neutrophil apoptosis, which was associated with the favored uptake of infected over uninfected neutrophils by DCs in the skin. More importantly, for the majority of infected DCs in the skin their initial encounter with the parasite occurred via capture of infected neutrophils, with a negative impact on CD4^+^ T cell priming.

These studies confirm the previous findings in *L. major* [5], [23], recently extended to *L. infantum* [24], that neutrophils are rapidly recruited to and accumulate in the inoculation site, and represent the predominant parasitized cell during the first 1–12 hours of infection in the skin. The inflammatory and infectious process induced by *L. major* in the skin may be regulated in a tissue specific manner, since recent observation by Gonçalves et al. [25] and confirmed by our own studies (data not shown) have revealed that when *L. major* metacyclics are introduced into the peritoneal cavity, neutrophils are neither the first infiltrating nor predominant infected cells. Our kinetic analysis of the *L. major* loaded dermis revealed that the rapid neutrophilic response is initiated in part by signals generated by the tissue injury produced by the needle injection itself, since a transient recruitment was observed in sham injected mice, and amplified by more durable signals derived from the parasite and/or from infected cells [26]. The fate of the infected neutrophils was followed by transfer of eGFP^hi^RFP^+^ cells into the ear dermis of C57BL/6 mice. By 4 hr, the majority of RFP^+^ cells recovered from the site were endogenous neutrophils and monocytes/macrophages that were eGFP**^−^**, consistent with our prior *in vivo* imaging results that readily captured infected neutrophils undergoing apoptosis and releasing viable parasites for subsequent uptake by other cells in the skin [5]. Thus, the ‘Trojan Horse’ hypothesis as originally proposed [17], in which neutrophils serve as a vector for silent entry of Leishmania into macrophages, has not been directly substantiated in these studies. We cannot, however, dismiss the possibility that phagosomal degradation of the eGFP signal occurred rapidly following engulfment of the infected neutrophils by macrophages. It is also possible that clearance of neutrophil-derived, apoptotic bodies by infected macrophages would still contribute to their deactivation and promote the intracellular survival and growth of the parasite, as proposed.

By contrast to macrophages, the evidence for the uptake of *L. major* infected neutrophils by DCs in the skin seems clear. Firstly, CD11c^+^ cells were the only endogenous cells associated with both the RFP and eGFP signals. Secondly, when the infections were initiated by RFP *L. major* metacyclics, the majority of the RFP^+^ DCs recovered from the injection site at 24 hr also stained positive for neutrophil-derived MPO and elastase. In studies by Ng et al. [27], two-photon imaging captured dermal DCs but not Langerhans cells taking up Leishmania promastigotes in the skin. We also found Langerin**^−^** dermal DCs as the major infected DC subset in the skin, but conclude based on their staining for neutrophil markers, and the absence of these markers in DCs that have taken up parasites in the absence of neutrophils, that the majority of the infected DCs acquired their parasites via engulfment of infected neutrophils. Favored uptake of infected over uninfected neutrophils was also observed, correlated with their accelerated expression of apoptotic markers that may have targeted them for more efficient recognition and clearance by DCs. Neutrophil ingestion of other microbial pathogens, notably *E. coli* [28], *Str. pneumoniae* [29], [30], *C. albicans* [31], *Sta. aureus* [32], and *M. tuberculosis* [33], has also been found to accelerate their apoptotic program. The findings involving *Leishmania* are inconsistent on this point, with delayed or enhanced expression of PtdSer observed on neutrophils obtained from human blood or the mouse peritoneal cavity and exposed to Leishmania *in vitro* [34]–[37]. The current studies are the first to compare the apoptotic profile of tissue infiltrated neutrophils that have taken up parasites, or not, in the inflamed dermis.

Apoptosis is an active process to regulate cellular homeostasis. Efferocytosis refers to the capture of apoptotic cells by phagocytes, primarily macrophages and immature DCs (iDC), and is itself thought to be a homeostatic mechanism to resolve inflammation and to maintain peripheral tolerance [13]. Recognition and engulfment of apoptotic cells, including apoptotic neutrophils, by DC is known to inhibit their production of pro-inflammatory cytokines, expression of costimulatory molecules, and their ability to stimulate T-cell proliferation [14], [38], [39]. The exploitation of these inhibitory signals by microbial pathogens is suggested by *in vitro* studies showing that *M. tuberculosis*-induced activation of human iDC can be inhibited by their co-culture with apoptotic neutrophils [40], and that *Plasmodium falciparum*-infected erythrocytes can inhibit the maturation of mouse DCs by binding to CD36, a known recognition receptor for apoptotic cells [41]. The present studies are the first to demonstrate efferocytosis involving neutrophils and DCs in an infection driven inflammatory setting *in vivo*. The sequestration of Leishmania antigens within apoptotic neutrophils would seem an especially efficient process to exploit the immunosuppressive signals conferred by the clearance of dying cells by DCs. Removing host neutrophils as a source of apoptotic cells was sufficient to reconstitute the immune function of infected DCs. It should be noted that in contrast to recent studies [42], we did not observe a reduction in either the total number of DCs nor infected DCs recovered from the ear following neutrophil depletion (Figure 5D and E). We would offer that while the prior study was confined to cells migrating out of the ear dermis ex vivo, our analysis was based on the greater recovery of cells following enzymatic digestion of the tissue. By comparing the *ex vivo* APC function of infected DCs recovered from the skin of mice depleted or not of neutrophils, and normalized for their RFP signals, the inhibitory effects of neutrophil uptake on DC maturation and Leishmania specific T cell activation could be formally demonstrated. The consequence of this inhibition in effectively delaying the onset of Leishmania specific T cell priming *in vivo* was directly supported by the enhanced, early OT-II priming to *Lm*-derived OVA in the neutrophil depleted mice.

The neutrophil - DC interactions that inhibit T cell priming following needle challenge with *L. major* might be relevant to more general vaccination protocols in which an acute neutrophilic infiltrate accumulates at the site of antigen deposition. A recent report by Yang et al. [43] described the negative influence of neutrophils on the T and B cell responses to protein antigens administered by needle in the footpad. It is clear, however, that apoptotic neutrophils can also provide a source of immunogenic molecules to DC, especially for cross-priming, and especially if accompanied by extrinsic maturation signals [44], [45]. The relative paucity of activation signals associated with the phagocytosis of Leishmania promastigotes by neutrophils is suggested by the fact that the parasite traffics to a non-lytic compartment, avoids activation of the NADPH oxidase, and survives capture by these cells [5], [37]. It should be noted that PtdSer exposure on the parasites themselves has been suggested to facilitate their silent entry into macrophages, [46], [47], and may be especially relevant to their initial survival in neutrophils. Following neutrophil depletion, or the resolution of the first wave of neutrophils in the site, the majority of the infected DCs recovered from the skin lacked neutrophil markers, and are presumed to have taken up the parasite directly. By contrast to the absence of activation signals associated with the direct uptake of *L. major* metacyclic promastigotes by macrophages, the activation of human and mouse DCs following their phagocytosis of these organisms *in vitro* is well described [48]. Direct uptake might allow for parasite antigens to be more accessible to the MHC class I and II processing machinery, for parasite encoded TLR agonists to more efficiently engage their respective receptors, and for activation pathways to proceed in the absence of the inhibitory signals induced by apoptotic cell clearance. By two weeks, the priming conditions had clearly improved, and neutrophil depletion did not further enhance the CD4^+^ T cell response, despite the reappearance of neutrophils in the site. In contrast to the initial wave, however, the infected neutrophils recovered at two weeks represented a small percentage of the total population of infected cells, and the majority of infected DCs no longer harbored neutrophil markers. It is likely that the conditions of neutrophil recruitment to and activation in the skin during the active stage of disease, possibly Th17 driven at this later time, are distinct from those associated with the acute infiltrate, and that the influence of these respective neutrophil populations on the anti-leishmanial response will also be distinct.

In the current studies, there was a significant difference in the effects of the neutrophil depleting antibodies, 1A8 and RB6-8C5, in potentiating the early OT-II response to infection with *Lm* SP-OVA in the skin. The 1A8 treatment critically confines the enhanced priming observed to specific depletion of Ly6G**^+^** neutrophils. The more powerful effects observed with the RB6-8C5 antibody is consistent with the more efficient depletion of neutrophils that was achieved, although the removal of an additional population of GR-1^+^ myeloid cells with suppressor activity [49], [50] cannot be discounted.

While our studies have employed a relatively high dose, needle challenge in order to recover a sufficient number of infected cells from the ear dermis for analysis, it should be emphasized that the initial wave of neutrophil recruitment to the infected sand fly bite site is more massive, localized, and sustained compared to the needle injection site [22]. This may explain why the ablation of the early neutrophilic response had such a strong effect in promoting protection against sand fly transmitted infection as compared to needle challenge [5], [51]–[53]. Thus, the impact of the early neutrophil - DC interactions described in these studies may be especially relevant to the inflammatory conditions elicited by natural sand fly transmission, as well as to that of other vector borne pathogens, in promoting the early establishment of infection and the progression of disease.

## Materials and Methods

### Mice

This study was carried out in strict accordance with the recommendations in the Guide for the Care and Use of Laboratory Animals of the National Institutes of Health. The protocol was approved by the Animal Care and Use Committee of the NIAID, NIH (protocol number LPD 68E). All mice were maintained at the NIAID animal care facility under specific pathogen-free conditions. Female C57BL/6 and B6.SJL congenic mice, and RAG1-deficient OT-II CD4^+^ TCR transgenic mice were purchased from Taconic Laboratories. C57BL/6 LYS-eGFP knock-in mice [18] were a gift from T. Graf (Albert Einstein University, NY) and were bred at Taconic Laboratories.

### *Leishmania major* parasites

Experiments were carried out using different lines of *L. majo*r*: L. major* Friedlin strain FV1 (MHOM/IL/80/FN); a stable transfected line of *L. major* FV1 promastigotes expressing a red fluorescent protein (*Lm*-RFP), *L. major* FV1 promastigotes expressing a portion of the ovalbumin gene encoding amino acids 139 to 386 containing the class II restricted epitope recognized by OT-II TCR transgenic CD4^+^ T cells (*Lm*- SP-OVA), and *L. major* V1- transfected with the control plasmid expressing *Leishmania donovani* 3′ nucleotidase-nuclease (*Lm*-NT). Transfected lines were generated as described previously [54]–[55].

### Parasite preparation and i.d. inoculation

Parasites were grown at 26°C in medium 199 supplemented with 20% heat-inactivated FCS (Gemini Bio-Products), 100 U/ml penicillin, 100 µg/ml streptomycin, 2 mM L-glutamine, 40 mM Hepes, 0.1 mM adenine (in 50 mM Hepes), 5 mg/ml hemin (in 50% triethanolamine), 1 mg/ml 6-biotin (M199/S), and 50 µg/ml of Geneticin (Gibco). Infective-stage, metacyclic promastigotes of *L. major* were isolated from stationary cultures (4–5 days old) by negative selection using peanut agglutinin (PNA, Vector Laboratories Inc). For flow cytometric studies of dermal and draining lymph node cells, mice were infected with the specified number of metacyclic promastigotes in the ear dermis by i.d. injection in a volume of 10 µl. In parallel, sham mice received i.d. injection of DMEM in a volume of 10 µl. To obtain chronically infected mice, animals were infected 16–20 weeks previously with 10^4^ *L. major* FV1 metacyclic promastigotes in the left hind footpad.

### Processing of ear tissue and dLN

Ear tissue was prepared as previously described [2]. Briefly, the two sheets of infected ear dermis were separated, deposited in DMEM containing 100 U/ml penicillin, 100 µg/ml streptomycin, and 0.2 mg/ml Liberase CI purified enzyme blend (Roche Diagnostics Corp.), and incubated for 1 h and 30 min at 37°C. Digested tissue was placed in a grinder and processed in a tissue homogenizer (Medimachine; Becton Dickenson). Retromaxillary (ear) lymph nodes were removed, and mechanically dissociated using tweezers and a syringe plunger. Tissue homogenates were filtered through a 70 µm cell strainer (Falcon Products).

### Immunolabeling and flow cytometry

Single-cell suspensions were incubated with an anti-Fc-γ III/II (CD16/32) receptor Ab (2.4G2, BD Biosciences) in RPMI without phenol red (Gibco) containing 1% FCS and stained with fluorochrome-conjugated antibodies. The following antibodies were used: APC- anti-mouse CD11c (HL3, BD Biosciences), PE-Cy7- anti-mouse CD11c (N418, eBioscience), PerCP-Cy5.5 or PE-Cy7- anti-mouse CD11b (M1/70, eBioscience); PerCP-Cy5.5- anti-mouse Ly6C (HK1.4, eBioscience); FITC- anti-mouse Ly6G (1A8, eBioscience); FITC- anti-mouse GR-1 (RB6-8C5, BD Biosciences); eFluor anti-mouse F4/80 (BM8, eBioscience), Alexafluor-700 anti-mouse MHC II (M5/114.15.2, eBioscience), APC- anti-mouse CD103 (M290, eBioscience), A488- anti-mouse Langerin (929F3.01, Dendritics), APC- anti-mouse CD40 (1C10, eBioscience), FITC- anti-mouse CD80 (16-10A1, eBioscience), PerCP-Cy5.5- anti-mouse CD86 (GL-1, BioLegend), APC- anti-mouse CD4 (RM4–5, eBioscience), PerCP-Cy5.5- anti-mouse CD45.2 (104, eBioscience); APC-eFluor 780 anti-mouse CD45.1 (A20, eBioscience), FITC- anti-mouse myeloperoxidase (MPO) (8F4, Hycult), anti-human neutrophil elastase (NE) (H-57, Santa Cruz), FITC conjugated using and amine reactive probe (Sigma-Aldrich). The isotype controls used (all obtained from BD Biosciences) were rat IgG1 (R3–34) and rat IgG2b (A95-1). The staining of surface and intracytoplasmic markers was performed sequentially: the cells were stained first for their surface markers, followed by a permeabilization step with BD Cytofix/Cytoperm (BD Biosciences) and staining for Langerin, MPO or NE. For intracellular detection of cytokines, cells were first stimulated with Leukocyte Activation Cocktail, plus GolgiPlug (BD Biosciences) according the manufacturers' instructions for 4 h in vitro. Following surface staining and permeabilization, cells were then stained with a combination of anti-mouse antibodies: PerCP-Cy5.5 anti-IL17A (eBio17B7, eBioscience) APC anti-IFN-g (XMG1.2, eBioscience), PE anti-IL-10 (JES5-16E3, BD Bioscience) in Perm/Wash buffer (BD Bioscience). Intracellular staining was carried out for 30 minutes on ice. The data were collected and analyzed using CELLQuest software and a FACScalibur or FacsDIVA software and a FacsCANTO flow cytometer (BD Biosciences). Neutrophils, dendritic cells, macrophages and monocytes from the ear dermis were identified based on size (forward scatter) and granularity (side scatter) and by surface phenotype as indicated in the text and figure legends.

### Cell purification, co-culture and adoptive transfer

Infected DCs (CD11c^+^RFP^+^) were purified using a FACSVantage or a FACsAria (BD Biosciences) cell sorter on cells recovered from the ear dermis 3 days after infection with 2×10^6^ *L. major-*RFP. For the analysis of the capacity of infected, dermal DCs to induce the secretion of IFNγ by *L. major* specific T cells, 4×10^4^ (Exp. 1) or 4.5×10^4^ (Exp. 2) infected dermal DCs pooled from 10 mice (20 ears) for each treatment group were co-cultured with 1×10^5^ T cells purified by negative selection (Miltenyi Biotec) from draining lymph nodes (dLNs) of B6 mice with a healed, primary infection with *L. major* FV1. After 3 days, culture supernatants were analyzed for IFN-γ production by ELISA (eBioscience). For adoptive transfer experiments, CD4^+^ T cells were purified from spleens and lymph nodes of RAG1-deficient OT-II CD4^+^ TCR transgenic mice by negative selection (Miltenyi Biotec). Purified CD4^+^ T cells were incubated at 2.5–5×10^7^ cells/ml in PBS with 0.5 µM CFSE (Invitrogen) for 10 min at 37°C. The reaction was stopped with 10% normal mouse serum, and the cells were washed twice with cold PBS/0.1% BSA. B6.SJL congenic mice received intravenously (i.v.) 2–5×10^5^ CFSE-labeled, purified CD4^+^ OT-II T cells either the same day or 14 days after challenge in the ear dermis with 10^5^ metacyclic promastigotes. Six days after adoptive transfer, the dLNs were removed and analyzed by flow cytometry. To obtain neutrophils recruited to the site of infection in the skin, LYS-eGFP mice were inoculated in the ear dermis with 2×10^6^ *Lm-*RFP. Twelve hours later the ear tissue was prepared as described above and infected (RFP^+^eGFP^hi^) and uninfected (RFP^−^eGFP^hi^) neutrophil populations were sorted from dermal tissue using a FACSVantage or a FACsAria (BD Biosciences) cell sorter. Sorted populations were washed once and immediately analyzed for apoptosis or injected into the ear dermis of C57BL/6 and B6SJL recipient mice in a volume of 10 ul.

### Assessment of PMN apoptosis

Sorted, infected (RFP^+^eGFP^hi^) and uninfected (RFP^−^eGFP^hi^) neutrophil populations were stained with Annexin-V-APC and 7-AAD (BD Biosciences) as recommended by the manufacturer. For TUNEL assays, neutrophil populations were fixed in 4% paraformaldehyde, and then labeled with the Beckman Coulter Mebstain Apoptosis kit using biotinylated dUTP. Cells were then incubated with streptavidin-conjugated APC (BD Pharmingen) for 30 min at room temperature. Cells were analyzed by flow cytometry.

### Neutrophil depletion

Neutrophils were depleted employing a single i.p. injection of 0.5 mg RB6-8C5 (anti-Gr-1), or 1 mg of 1A8 (anti-Ly6G, BioXCell), or GL113 (control IgG, BioXCell), 1 d prior to parasite injection. The efficiency and specificity of the depletions were evaluated on dermal cell preparations, and on heparinized whole blood.

### Statistical analysis

Statistical significance between groups was determined by the unpaired, two-tailed student's *t* test using Prism software (GraphPad).

## Supporting Information

Figure S1 Dermal DCs taking up infected neutrophils from congenic donors are of host origin. (A) Representative dot plot of gated RFP^+^ dermal cells recovered from a single ear of a B6.SJL (host, CD45.1) mouse 4 hr after i.d. injection of 2.5×10^4^ RFP^+^ eGFP^hi^ neutrophils (donor, CD45.2), and analyzed for their expression of eGFP and CD11c. (B) Representative histogram plots of CD45.1 stained GFP^hi^CD11c^−^ neutrophils (gray filled), GFP^+^CD11c^+^ DCs (black line) and GFP^−^CD11c^−^ cells (gray line).
(TIF)

Figure S2 Expression of MPO by leukocyte subsets in the skin. Histogram plots of MPO stained, RFP**^−^** DCs (black filled), RFP**^+^** DCs (thin black line), inflammatory monocytes (grey filled), and neutrophils (thick gray line), recovered from the ear dermis 24 hr post-infection with 2×10^5^ *Lm*-RFP parasites.
(TIF)

Figure S3 Expression of NE by infected DCs in the skin. Mice were treated with GL113, RB6-8C5 or 1A8 mAb 24 hr before infection in the ear dermis with 2×10^5^ *Lm-*RFP. Histogram plots of RFP**^−^** (gray filled) and RFP**^+^** (black line) DCs recovered from the ear dermis 24 hr after infection and stained for NE. Data are representative of 3 independent experiments.
(TIF)

Figure S4 CD11b^hi^Langerin**^−^**CD103**^−^** DCs harbor Leishmania parasites. Single cell suspensions were prepared from the ear dermis 24 hr post-infection with 2×10^5^ *Lm*-RFP parasites. DCs (CD11c**^+^**MHCII**^+^**) were gated as Langerin**^+^**CD103**^−^** (region 1), Langerin**^+^**CD103**^+^** (region 2), and Langerin**^−^**CD103**^−^** (region 3). DC subpopulations were analyzed with respect to CD11b expression and RFP signal. Data are representative of 2 independent experiments.
(TIF)

## References

[1] Sacks D, Anderson C (2004). Re-examination of the immunosuppressive mechanisms mediating non-cure of Leishmania infection in mice.. Immunol Rev.

[2] Belkaid Y, Mendez S, Lira R, Kadambi N, Milon G (2000). A natural model of Leishmania major infection reveals a prolonged “silent” phase of parasite amplification in the skin before the onset of lesion formation and immunity.. J Immunol.

[3] Laskay T, van Zandbergen G, Solbach W (2008). Neutrophil granulocytes as host cells and transport vehicles for intracellular pathogens: apoptosis as infection-promoting factor.. Immunobiology.

[4] Peters NC, Sacks DL (2009). The impact of vector-mediated neutrophil recruitment on cutaneous leishmaniasis.. Cell Microbiol.

[5] Peters NC, Egen JG, Secundino N, Debrabant A, Kimblin N (2008). In vivo imaging reveals an essential role for neutrophils in leishmaniasis transmitted by sand flies.. Science.

[6] Kennedy AD, DeLeo FR (2009). Neutrophil apoptosis and the resolution of infection.. Immunol Res.

[7] Nauseef WM (2007). How human neutrophils kill and degrade microbes: an integrated view.. Immunol Rev.

[8] Scapini P, Lapinet-Vera JA, Gasperini S, Calzetti F, Bazzoni F (2000). The neutrophil as a cellular source of chemokines.. Immunol Rev.

[9] Yang D, Chen Q, Chertov O, Oppenheim JJ (2000). Human neutrophil defensins selectively chemoattract naive T and immature dendritic cells.. J Leukoc Biol.

[10] Megiovanni AM, Sanchez F, Robledo-Sarmiento M, Morel C, Gluckman JC (2006). Polymorphonuclear neutrophils deliver activation signals and antigenic molecules to dendritic cells: a new link between leukocytes upstream of T lymphocytes.. J Leukoc Biol.

[11] Blomgran R, Ernst JD (2011). Lung Neutrophils Facilitate Activation of Naive Antigen-Specific CD4+ T Cells during Mycobacterium tuberculosis Infection.. J Immunol.

[12] Sauter B, Albert ML, Francisco L, Larsson M, Somersan S (2000). Consequences of cell death: exposure to necrotic tumor cells, but not primary tissue cells or apoptotic cells, induces the maturation of immunostimulatory dendritic cells.. J Exp Med.

[13] Steinman RM, Turley S, Mellman I, Inaba K (2000). The induction of tolerance by dendritic cells that have captured apoptotic cells.. J Exp Med.

[14] Stuart LM, Lucas M, Simpson C, Lamb J, Savill J (2002). Inhibitory effects of apoptotic cell ingestion upon endotoxin-driven myeloid dendritic cell maturation.. J Immunol.

[15] Leon B, Lopez-Bravo M, Ardavin C (2007). Monocyte-derived dendritic cells formed at the infection site control the induction of protective T helper 1 responses against Leishmania.. Immunity.

[16] Laskay T, van Zandbergen G, Solbach W (2003). Neutrophil granulocytes–Trojan horses for Leishmania major and other intracellular microbes?. Trends Microbiol.

[17] van Zandbergen G, Klinger M, Mueller A, Dannenberg S, Gebert A (2004). Cutting Edge: Neutrophil Granulocyte Serves as a Vector for Leishmania Entry into Macrophages.. J Immunol.

[18] Faust N, Varas F, Kelly LM, Heck S, Graf T (2000). Insertion of enhanced green fluorescent protein into the lysozyme gene creates mice with green fluorescent granulocytes and macrophages.. Blood.

[19] Egan CE, Sukhumavasi W, Bierly AL, Denkers EY (2008). Understanding the multiple functions of Gr-1(+) cell subpopulations during microbial infection.. Immunol Res.

[20] Wojtasiak M, Pickett DL, Tate MD, Londrigan SL, Bedoui S (2010). Depletion of Gr-1+, but not Ly6G+, immune cells exacerbates virus replication and disease in an intranasal model of herpes simplex virus type 1 infection.. J Gen Virol.

[21] Merad M, Ginhoux F, Collin M (2008). Origin, homeostasis and function of Langerhans cells and other langerin-expressing dendritic cells.. Nat Rev Immunol.

[22] Peters NC, Kimblin N, Secundino N, Kamhawi S, Lawyer P (2009). Vector transmission of leishmania abrogates vaccine-induced protective immunity.. PLoS Pathog.

[23] Beil WJ, Meinardus-Hager G, Neugebauer DC, Sorg C (1992). Differences in the onset of the inflammatory response to cutaneous leishmaniasis in resistant and susceptible mice.. J Leukoc Biol.

[24] Thalhofer CJ, Chen Y, Sudan B, Love-Homan L, Wilson ME (2011). Leukocytes infiltrate the skin and draining lymph nodes in response to the protozoan Leishmania infantum chagasi.. Infect Immun.

[25] Goncalves R, Zhang X, Cohen H, Debrabant A, Mosser DM (2011). Platelet activation attracts a subpopulation of effector monocytes to sites of Leishmania major infection.. J Exp Med.

[26] van Zandbergen G, Hermann N, Laufs H, Solbach W, Laskay T (2002). Leishmania promastigotes release a granulocyte chemotactic factor and induce interleukin-8 release but inhibit gamma interferon-inducible protein 10 production by neutrophil granulocytes.. Infect Immun.

[27] Ng LG, Hsu A, Mandell MA, Roediger B, Hoeller C (2008). Migratory dermal dendritic cells act as rapid sensors of protozoan parasites.. PLoS Pathog.

[28] Watson RW, Redmond HP, Wang JH, Condron C, Bouchier-Hayes D (1996). Neutrophils undergo apoptosis following ingestion of Escherichia coli.. J Immunol.

[29] Zysk G, Bejo L, Schneider-Wald BK, Nau R, Heinz H (2000). Induction of necrosis and apoptosis of neutrophil granulocytes by Streptococcus pneumoniae.. Clin Exp Immunol.

[30] Engelich G, White M, Hartshorn KL (2001). Neutrophil survival is markedly reduced by incubation with influenza virus and Streptococcus pneumoniae: role of respiratory burst.. J Leukoc Biol.

[31] Rotstein D, Parodo J, Taneja R, Marshall JC (2000). Phagocytosis of Candida albicans induces apoptosis of human neutrophils.. Shock.

[32] Yamamoto A, Taniuchi S, Tsuji S, Hasui M, Kobayashi Y (2002). Role of reactive oxygen species in neutrophil apoptosis following ingestion of heat-killed Staphylococcus aureus.. Clin Exp Immunol.

[33] Perskvist N, Long M, Stendahl O, Zheng L (2002). Mycobacterium tuberculosis promotes apoptosis in human neutrophils by activating caspase-3 and altering expression of Bax/Bcl-xL via an oxygen-dependent pathway.. J Immunol.

[34] Aga E, Katschinski DM, van Zandbergen G, Laufs H, Hansen B (2002). Inhibition of the spontaneous apoptosis of neutrophil granulocytes by the intracellular parasite Leishmania major.. J Immunol.

[35] Charmoy M, Auderset F, Allenbach C, Tacchini-Cottier F (2010). The prominent role of neutrophils during the initial phase of infection by Leishmania parasites.. J Biomed Biotechnol.

[36] Xin L, Vargas-Inchaustegui DA, Raimer SS, Kelly BC, Hu J (2010). Type I IFN receptor regulates neutrophil functions and innate immunity to Leishmania parasites.. J Immunol.

[37] Gueirard P, Laplante A, Rondeau C, Milon G, Desjardins M (2008). Trafficking of Leishmania donovani promastigotes in non-lytic compartments in neutrophils enables the subsequent transfer of parasites to macrophages.. Cell Microbiol.

[38] Savill J, Dransfield I, Gregory C, Haslett C (2002). A blast from the past: clearance of apoptotic cells regulates immune responses.. Nat Rev Immunol.

[39] Clayton AR, Prue RL, Harper L, Drayson MT, Savage CO (2003). Dendritic cell uptake of human apoptotic and necrotic neutrophils inhibits CD40, CD80, and CD86 expression and reduces allogeneic T cell responses: relevance to systemic vasculitis.. Arthritis Rheum.

[40] Aleman M, de la Barrera S, Schierloh P, Yokobori N, Baldini M (2007). Spontaneous or Mycobacterium tuberculosis-induced apoptotic neutrophils exert opposite effects on the dendritic cell-mediated immune response.. Eur J Immunol.

[41] Urban BC, Willcox N, Roberts DJ (2001). A role for CD36 in the regulation of dendritic cell function.. Proc Natl Acad Sci U S A.

[42] Charmoy M, Brunner-Agten S, Aebischer D, Auderset F, Launois P (2010). Neutrophil-derived CCL3 is essential for the rapid recruitment of dendritic cells to the site of Leishmania major inoculation in resistant mice.. PLoS Pathog.

[43] Yang CW, Strong BS, Miller MJ, Unanue ER (2010). Neutrophils influence the level of antigen presentation during the immune response to protein antigens in adjuvants.. J Immunol.

[44] Xiaoxiao W, Sibiao Y, Xiaopeng X, Ping Z, Gang C (2007). Neutrophils induce the maturation of immature dendritic cells: a regulatory role of neutrophils in adaptive immune responses.. Immunol Invest.

[45] Bennouna S, Bliss SK, Curiel TJ, Denkers EY (2003). Cross-talk in the innate immune system: neutrophils instruct recruitment and activation of dendritic cells during microbial infection.. J Immunol.

[46] Wanderley JLM, Moreira MEC, Benjamin A, Bonomo AC, Barcinski MA (2006). Mimicry of Apoptotic Cells by Exposing Phosphatidylserine Participates in the Establishment of Amastigotes of Leishmania (L) amazonensis in Mammalian Hosts.. J Immunol.

[47] van Zandbergen G, Bollinger A, Wenzel A, Kamhawi S, Voll R (2006). Leishmania disease development depends on the presence of apoptotic promastigotes in the virulent inoculum.. Proc Natl Acad Sci U S A.

[48] Soong L (2008). Modulation of dendritic cell function by Leishmania parasites.. J Immunol.

[49] Van Ginderachter JA, Beschin A, De Baetselier P, Raes G (2010). Myeloid-derived suppressor cells in parasitic infections.. Eur J Immunol.

[50] Gabrilovich DI, Nagaraj S (2009). Myeloid-derived suppressor cells as regulators of the immune system.. Nat Rev Immunol.

[51] Chen L, Zhang ZH, Watanabe T, Yamashita T, Kobayakawa T (2005). The involvement of neutrophils in the resistance to Leishmania major infection in susceptible but not in resistant mice.. Parasitol Int.

[52] Ribeiro-Gomes FL, Otero AC, Gomes NA, Moniz-De-Souza MC, Cysne-Finkelstein L (2004). Macrophage interactions with neutrophils regulate Leishmania major infection.. J Immunol.

[53] Tacchini-Cottier F, Zweifel C, Belkaid Y, Mukankundiye C, Vasei M (2000). An immunomodulatory function for neutrophils during the induction of a CD4+ Th2 response in BALB/c mice infected with Leishmania major.. J Immunol.

[54] Kimblin N, Peters N, Debrabant A, Secundino N, Egen J (2008). Quantification of the infectious dose of Leishmania major transmitted to the skin by single sand flies.. Proc Natl Acad Sci U S A.

[55] Bertholet S, Debrabant A, Afrin F, Caler E, Mendez S (2005). Antigen requirements for efficient priming of CD8+ T cells by Leishmania major-infected dendritic cells.. Infect Immun.

