# Supplementary figures and images for: Efficient Capture of Infected Neutrophils by Dendritic Cells in the Skin Inhibits the Early Anti-Leishmania Response
Source: PLoS Pathog. 2012 Feb 16;8(2):e1002536. doi: 10.1371/journal.ppat.1002536 (PMC3280984; doi:10.1371/journal.ppat.1002536)

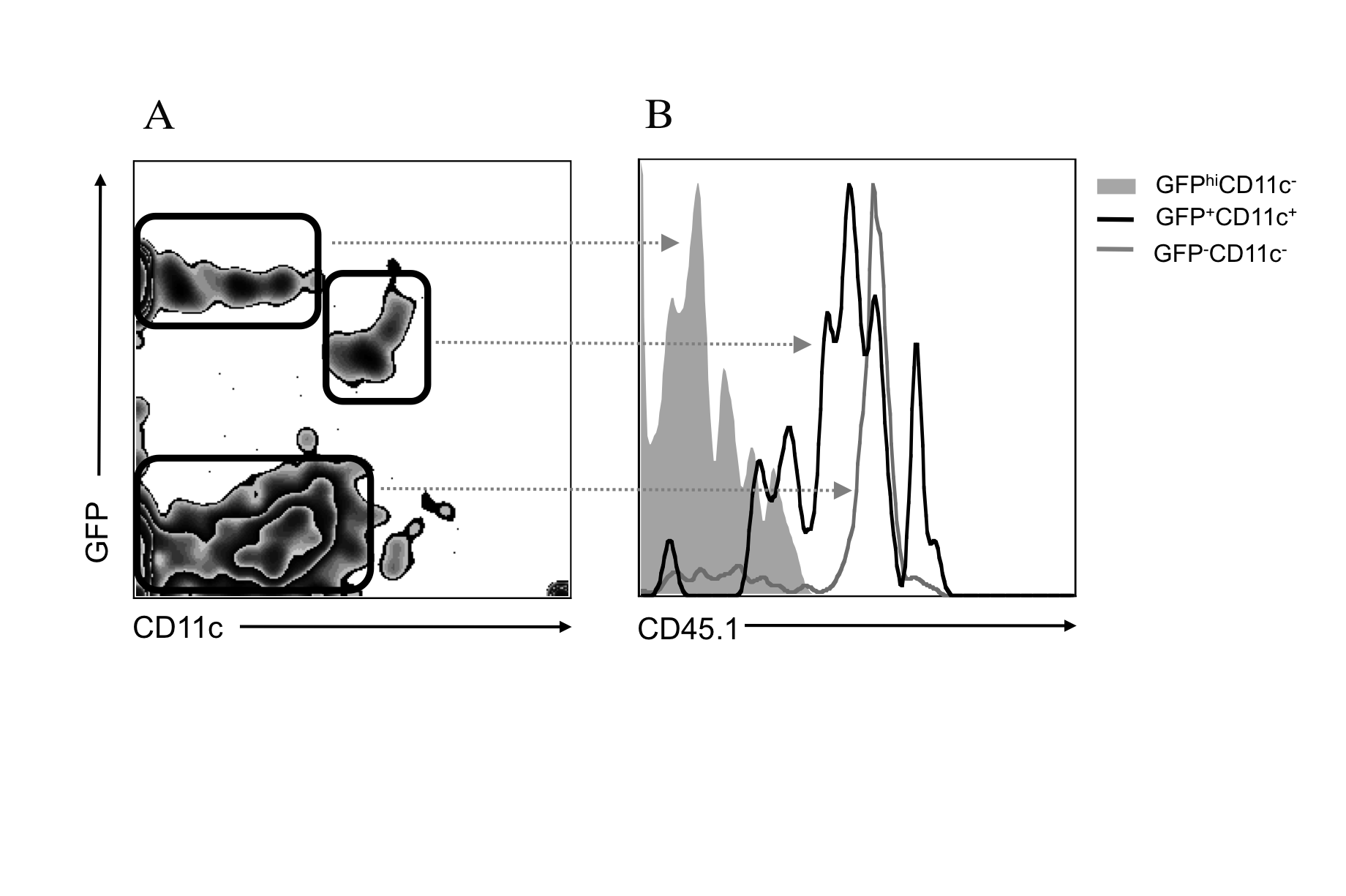

Supplement: Figure S1 — Dermal DCs taking up infected neutrophils from congenic donors are of host origin. (A) Representative dot plot of gated RFP+ dermal cells recovered from a single ear of a B6.SJL (host, CD45.1) mouse 4 hr after i.d. injection of 2.5×10^4^ RFP+ eGFPhi neutrophils (donor, CD45.2), and analyzed for their expression of eGFP and CD11c. (B) Representative histogram plots of CD45.1 stained GFPhiCD11c− neutrophils (gray filled), GFP+CD11c+ DCs (black line) and GFP−CD11c− cells (gray line). (TIF) [file ppat.1002536.s001.tif]

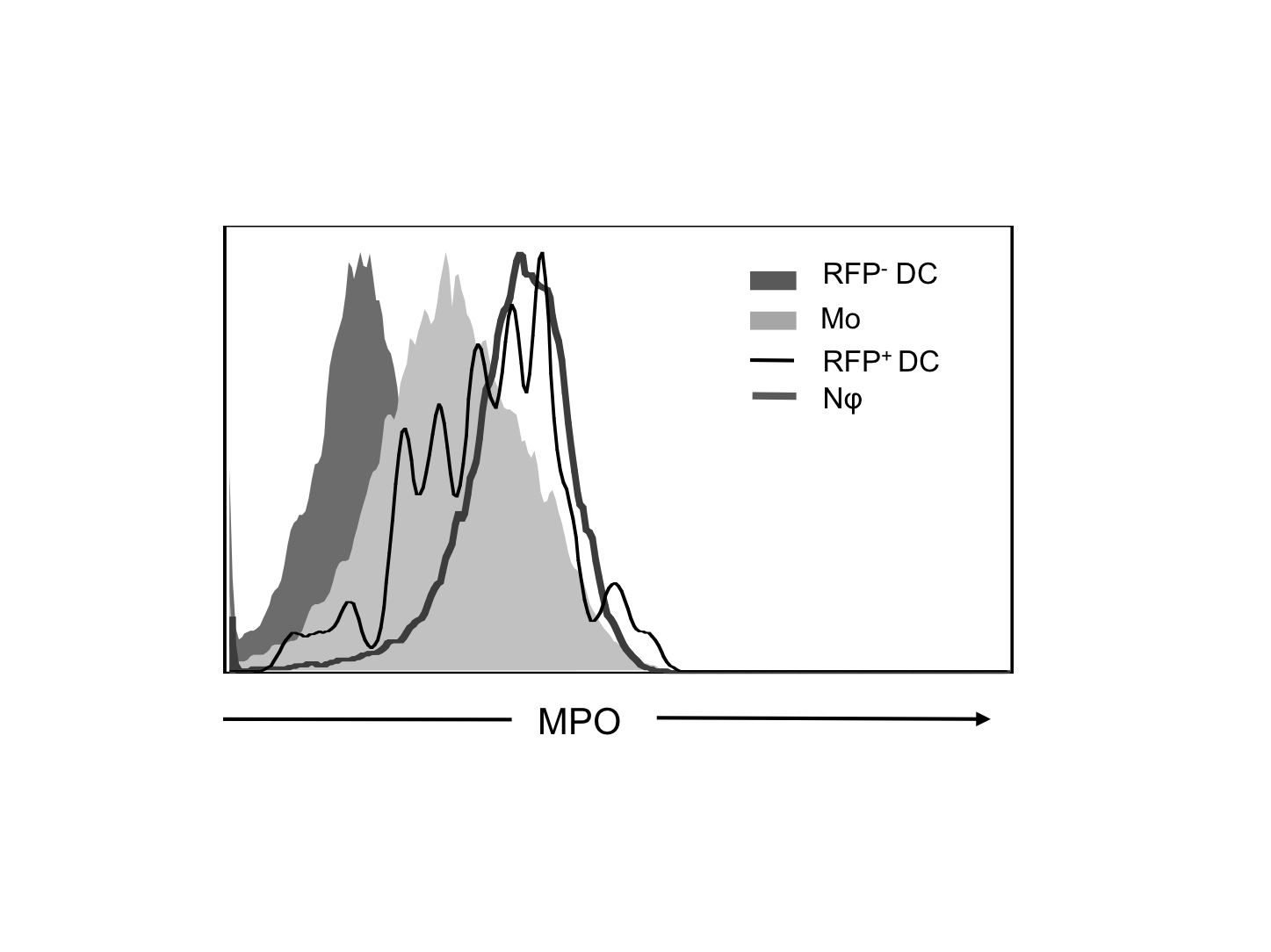

Supplement: Figure S2 — Expression of MPO by leukocyte subsets in the skin. Histogram plots of MPO stained, RFP− DCs (black filled), RFP+ DCs (thin black line), inflammatory monocytes (grey filled), and neutrophils (thick gray line), recovered from the ear dermis 24 hr post-infection with 2×10^5^ Lm-RFP parasites. (TIF) [file ppat.1002536.s002.tif]

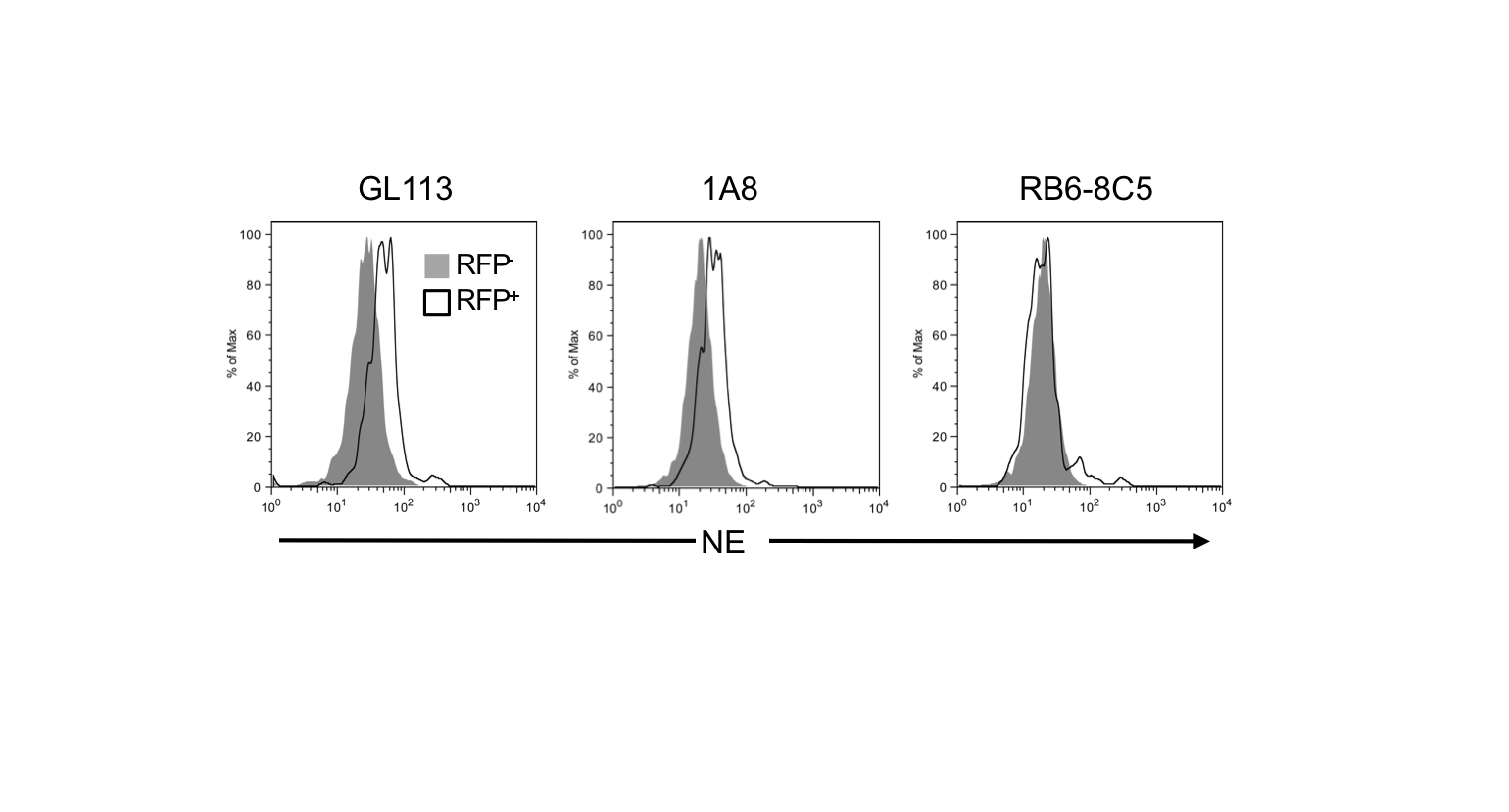

Supplement: Figure S3 — Expression of NE by infected DCs in the skin. Mice were treated with GL113, RB6-8C5 or 1A8 mAb 24 hr before infection in the ear dermis with 2×10^5^ Lm-RFP. Histogram plots of RFP− (gray filled) and RFP+ (black line) DCs recovered from the ear dermis 24 hr after infection and stained for NE. Data are representative of 3 independent experiments. (TIF) [file ppat.1002536.s003.tif]

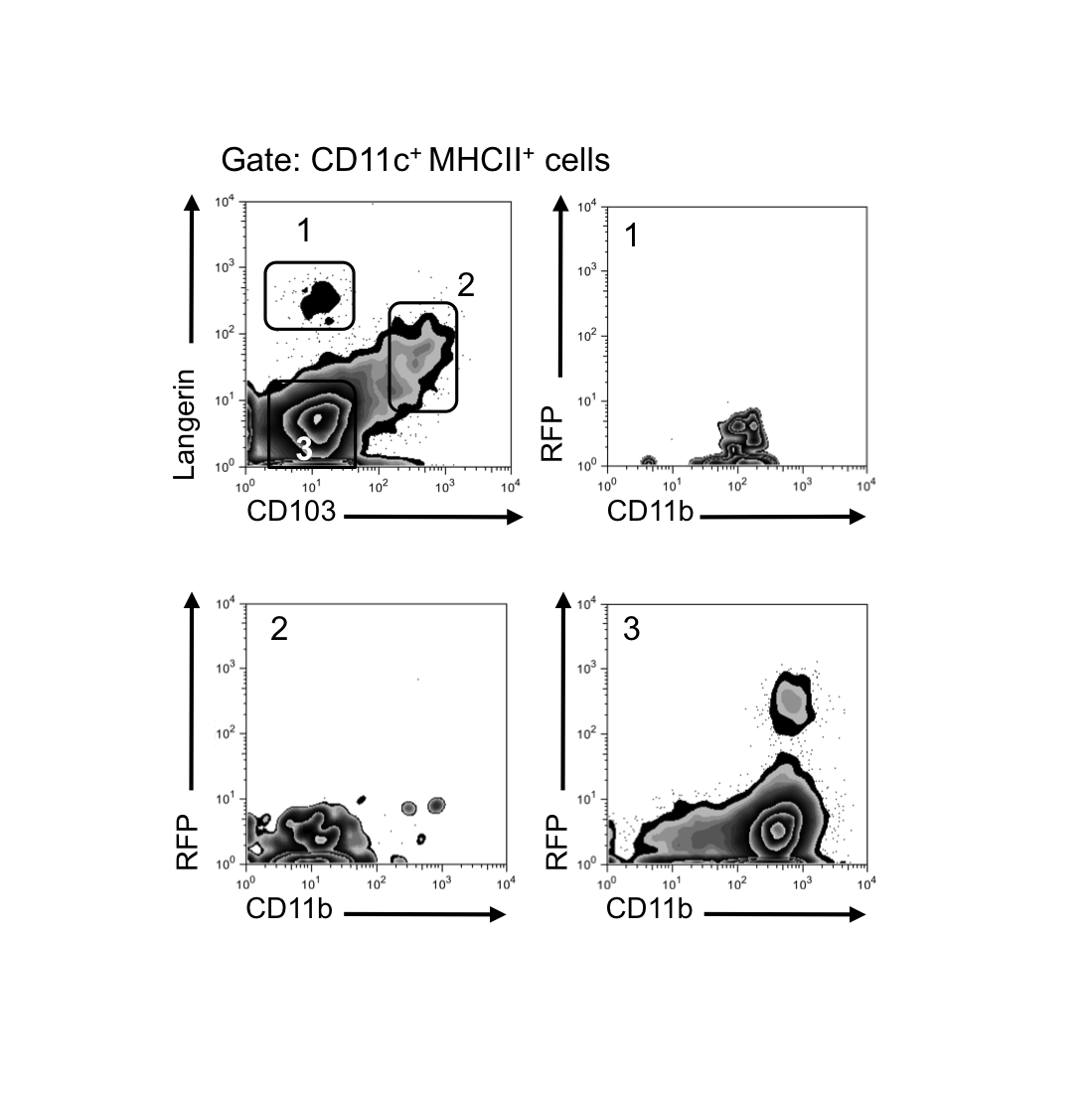

Supplement: Figure S4 — CD11bhiLangerin−CD103− DCs harbor Leishmania parasites. Single cell suspensions were prepared from the ear dermis 24 hr post-infection with 2×10^5^ Lm-RFP parasites. DCs (CD11c+MHCII+) were gated as Langerin+CD103− (region 1), Langerin+CD103+ (region 2), and Langerin−CD103− (region 3). DC subpopulations were analyzed with respect to CD11b expression and RFP signal. Data are representative of 2 independent experiments. (TIF) [file ppat.1002536.s004.tif]
